# Foreign Summary

**Published:** 1840-02-15

**Authors:** 


### FOREIGN SUMMARY.

*Preservation of subjects for dissection.*—After a variety of experiments, MM. Babington and Rees have succeeded in preserving subjects for anatomical purposes, by injecting the vascular system with pyroxylic spirit. The following account shows how well this substance is calculated for the purposes to which it was applied :—

On the 15th of May last, a convict at Woolwich, twenty-three years of age, died of inflammation of the bowels ; and, on the 18th, his body was sent to Guy's Hospital for dissection. It was neither œdematous, nor in a state of decomposition, and was in a fair condition for anatomical purposes. On the 21st, a gallon of pyroxylic spirit was injected into the aorta, and the body was placed in a water-tight shell, or trough, made of slate, and loosely covered with a wooden lid. This trough was deposited in a cellar, the stone floor of which was about two feet below the surface of the ground. On the 29th, the lid was removed, for the first time, and the body was found to be perfectly fresh. From the 29th of May, to the 12th of June, the subject was examined, by removing the lid of the trough every two or three days, and no change was perceptible until the latter date. At that time the only sign of alteration was the appearance of two or three brown streaks—evidently veins—on the inside of the thighs, and a separation of the cuticle of the hands from the true skin, which began to assume a greenish hue. Every other part of the body was perfectly preserved, and of a natural colour. There was no putrid odour on opening the lid of the trough, but the characteristic smell of the pyroxylic spirit was in some measure passing off. An incision into the middle of the right thigh, showed that the fat, muscles, blood-vessels and nerves were in a complete state of preservation. It should be observed, that ever since the injection of the subject, the weather had been that of established summer ; and that a second body, received from Woolwich, was so decomposed in three days after its arrival, as to be totally unfit for dissection. On the last examination, as well as on two or three previous occasions, fluid was observed to occupy the bottom of the trough, and this it was thought advisable to remove ; it was likewise determined to throw another quart of pyroxylic spirit into the aorta.

On the 24th of June, the body was removed to the dissecting-room, and placed on the table, for the purpose of being thoroughly dissected. With the exception of a greenish appearance on the outer part of the left thigh, and the brown streaks already mentioned, it appeared, when brought into the light, perfectly preserved. The skin on the back of the hands, instead of putrifying, had dried, and become transparent; while the greenness of the left thigh proved, on incision, to be quite superficial. The dissection was undertaken by eight gentlemen, and completed by the 13th of July; and it is testified by them all, that every anatomical purpose was as fully answered as if the subject had been quite recent. The various parts, on being laid open, were of natural colour, and of firm texture. The tendons and ligaments were silvery and white, and the nerves had lost none of their tenacity. The pectoral muscles alone formed an exception to the natural colour which was elsewhere maintained ; this appeared to be attributable to the macerating effects of a wetted cloth that had been laid upon the breast, to prevent evaporation through the aperture, by which the injection had been accomplished. The parts which were exposed by dissection gradually dried, changing, in the course of a day or two, to a dark colour, and, instead of putrifying, becoming hard. The brain, although it had retained its form, was soft, semi-putrid and unfit for demonstration : it must be borne in mind, however, that had the head been opened six days after death—at which period the subject was injected—this probably would have been the case.

The advantages of employing pyroxylic spirit are, 1st, its extreme fluidity, in consequence of which it may be thrown into the minutest vessels. 2dly, its freedom from colour. 3dly, its cheapness; for a gallon is sufficient to inject a full sized subject; and even with the present limited manufacture of it, it is only half the price of alcohol ; while it possesses infinitely greater antiseptic powers, and is, in common with that fluid, miscible with water, in all proportions. 4thly, its innocuous nature, and its freedom from any corrosive action upon steel instruments.—*Lancet, abridged from Guy's Hospital Reports, No.* 9.

---

*Comparative digestibility of food.*—We must not determine upon the digestibility, or at least the nourishing properties of food, from the feeling of facility with which it is removed from the stomach. All those kinds of food which, by strongly exciting the motion of the stomach, go quickly into the intestines, may be easily borne by the stomach, but are very imperfectly dissolved, and at last are followed by all the consequences of disturbed digestion. They afford the body, therefore, only a very small quantity of digestible matter, and are again excreted mostly unchanged. On the contrary, there are other kinds of food which, on account of their long continuance in the stomach, appear for the moment to be hard to digest, yet are nevertheless perfectly digestible, and never followed by bad consequences upon passing into the rest of the intestines.—*Prof. Schultz on Digestion.*

